# DDT Paradox

**DOI:** 10.1289/ehp.1103957

**Published:** 2011-10-01

**Authors:** Richard Tren, Donald Roberts

**Affiliations:** Africa Fighting Malaria, Washington, DC, E-mail: rtren@fightingmalaria.org; Uniformed Services University of the Health Sciences, Bethesda, Maryland

Bouwman et al. (2011) characterized anti-DDT, centrist-DDT and pro-DDT positions, and stated that they “could find no current outright anti-DDT activities.” This conclusion is false and misleading.

Several activist groups currently promote an anti-DDT agenda, routinely hyping supposed human health and environmental harm from DDT and ignoring studies that find no association between DDT and such harm. For instance, the description of Biovision’s “Stop DDT” project states that “Biovision is engaged to achieve a world-wide ban on DDT” (Biovision 2011). Such a statement could be ignored if it were not for the fact that Hans Herren, president of Biovision, was a member of the Stockholm Convention’s DDT Expert Group, as were two of the authors of Bouwman et al. (2011)—Bouwman and van den Berg. Furthermore, Bouwman et al. ignored the Secretariat of the Stockholm Convention’s promotion of an arbitrary deadline for cessation of DDT production by 2020 (United Nations Environment Programme 2007). The Secretariat’s promotion of this deadline undermines use and production of DDT and is *ultra vires,* because the convention excludes any deadline.

In identifying the “pro-DDT” faction,  Bouwman et al. (2011) attempted to characterize it as a minority view while ignoring national malaria control programs and ministers of health who repeatedly proclaim the importance of DDT for disease control programs in countries with high incidence of malaria. Indeed, the Southern African Development Community (SADC) Ministers of Health agreed at their November 2010 meeting that DDT was still required (SADC 2011). In addition, at the recent fifth meeting of the Conference of Parties to the Stockholm Convention, Namibia and the SADC announced their intention to produce DDT locally (SADC 2011). Furthermore, the 35 heads of state and government who are members of the African Leaders Malaria Alliance (ALMA) recently endorsed use of DDT in indoor residual spraying (IRS) (ALMA 2010). Such organized actions by affected countries bespeak broad recognition of scientific issues and continuing need for DDT in malaria control programs. Those actions expose the misrepresentations of those who contend support for DDT is limited to a small number of extremists.

Bouwman et al. (2011) argued that “evidence of adverse health effects due to DDT … is mounting” and therefore DDT should be accompanied by information on the potential side effects, just as with prescription medicine. We believe that the interpretation of the mounting evidence is itself a minority view and that their argument is false.

The World Health Organization’s (WHO) review of human health aspects of DDT use in IRS concluded that “for households where IRS is undertaken, there was a wide range of DDT and DDE serum levels between studies. Generally, these levels are below potential levels of concern for populations” (WHO 2011). None of the thousands of studies that have been conducted regarding possible human health effects of DDT satisfy even the most basic epidemiological criteria to prove a cause-and-effect relationship. In their commentary, Bouwman et al. (2011) confused a large number of studies that uniformly fail the criterion of consistency in demonstrating that DDT causes actual harm, with isolated studies revealing some statistical association or correlation as a suggestion of harm. It is on this basis that the authors argued for precaution in the use of DDT. In contrast, we argue that precaution should govern Bouwman et al.’s aggressive anti-DDT campaigning and not precaution in the use of DDT to prevent disease and save lives. The growing number of studies is not proof or evidence that DDT causes harm, but it is evidence of growing funding for research on this topic.

Bouwman et al. (2011) argued that households should be informed about unproven and speculative risks from DDT. Their argument must be rejected as the worst form of scaremongering because it will result in growing risk of disease and death from malaria while providing no proven health benefit. Ignoring proven and catastrophic health decrements from malaria infections while warning of theoretical concerns about DDT exposures is a function of ideology. Such precautionary messaging is not good public health policy or sound science.

## References

[r1] ALMA (African Leaders Malaria Alliance) (2010).

[r2] Biovision (2011). Projects: International. Stop DDT: Promoting Effective and Environmentally Sound Alternatives to DDT.. http://www.biovision.ch/fileadmin/pdf/e/projects/2011/International/BV_Factsheet_Stop_DDT_2011_E.pdf.

[r3] Bouwman H van den Berg H Kylin H. 2011 DDT and malaria prevention: addressing the paradox. Environ Health Perspect 119 744 747; doi:10.1289/ehp.1002127 [Online 18 January 2011] 21245017 PMC3114806

[r4] SADC (Southern African Development Community) (2011). Letter from SADC to A Steiner, Executive Director, United Nations Environment Programme, Notification of SADC Countries Respecting DDT Use and Production, 5 April 2011.. http://www.fightingmalaria.org/pdfs/sadclettertounep.pdf.

[r5] United Nations Environment Programme (2007). Future Plans for Work on DDT Elimination. A Stockholm Convention Secretariat Position Paper, November 2007. Geneva: Secretariat of the Stockholm Convention.. http://chm.pops.int/Portals/0/Repository/DDT-general/UNEP-POPS-DDT-PROP-SSCPP.English.PDF.

[r6] WHO (World Health Organization) (2011). DDT in Indoor Residual Spraying: Human Health Aspects. Environmental Health Criteria 241. Geneva:WHO.. http://www.who.int/ipcs/publications/ehc/ehc241.pdf.

